# Editorial: Investigating the roles of nutritional determinants, genetic predispositions, and environmental risk factors in the development of obesity and associated metabolic disorders

**DOI:** 10.3389/fnut.2026.1827168

**Published:** 2026-03-27

**Authors:** Liwan Fu, Haibo Li, Jingcheng Yang

**Affiliations:** 1 Center for Non-communicable Disease Management, Beijing Children's Hospital, Capital Medical University, National Center for Children's Health, Beijing, China; 2 Fujian Maternity and Child Health Hospital, College of Clinical Medicine for Obstetrics and Gynecology and Pediatrics, Fujian Medical University, Fuzhou, China; 3 Computational Research Center for Complex Chronic Diseases, Massachusetts General Hospital and Harvard Medical School, Boston, MA, United States

**Keywords:** biomarkers, gene-environment interactions, metabolic disorders, nutritional determinants, obesity, precision prevention

Obesity has evolved into one of the most formidable public health challenges of the 21st century (1), serving as a gateway to a spectrum of metabolic diseases including type 2 diabetes, cardiovascular disorders, and certain cancers (2). The pathogenesis of obesity is neither linear nor monolithic; rather, it emerges from an intricate web of nutritional determinants, genetic predispositions, and environmental risk factors that collectively dysregulate energy homeostasis and metabolic pathways (3–7). This Research Topic was conceived to dissect these complex interactions and to illuminate the mechanisms by which they converge to promote metabolic dysfunction. The 10 articles published in this Research Topic collectively advance our understanding of these multifaceted relationships, offering novel insights that span from fundamental mechanisms to clinical applications.

Several contributions to this Research Topic underscore the critical importance of refining how we measure and conceptualize adiposity. The study by Cai et al. introduces the Chinese Visceral Adiposity Index (CVAI) as a robust tool for identifying gout risk in patients with type 2 diabetes, demonstrating that ethnicity-specific indices of visceral fat outperform traditional anthropometric measures. This theme of precise phenotyping is extended by Chen, Liang et al., who evaluate the CHG index—a composite of cholesterol, HDL-C, and glucose—for identifying metabolic dysfunction-associated steatotic liver disease (MASLD) in non-obese Asian populations, showing superior discriminative ability compared to conventional single markers. Similarly, Wan et al. examine the association between body mass index and left ventricular ejection fraction in obese patients with atrial fibrillation, revealing that higher BMI correlates with reduced cardiac contractile function independent of left atrial volume. These studies collectively argue for moving beyond BMI as a standalone metric toward more nuanced, multi-dimensional assessments of metabolic health (8, 9).

The developmental origins of metabolic risk receive compelling attention in this Research Topic. Chen, Miao et al., in a prospective birth cohort of over 20,000 Chinese women, demonstrate that metabolically unhealthy phenotypes—whether in normal-weight or overweight individuals—significantly increase preterm birth risk, with additive interactions between metabolic abnormalities and overweight status. This work has profound implications for preconception and prenatal care, suggesting that metabolic screening should extend beyond weight alone (10). Complementing this population-level perspective, Xie et al. investigate metabolically unhealthy obesity in children, identifying fat mass, visceral fat, and fat-free mass as independent predictors, and revealing that lifestyle factors such as fast food consumption and sleep duration interact with body composition to shape metabolic outcomes. These findings emphasize that childhood represents a critical window for intervention, where modifiable behaviors can attenuate genetic and metabolic risks.

The interplay between genetic susceptibility and environmental exposures emerges as a central theme. Mao et al. provide elegant evidence from a pregnancy cohort that the protective effect of the *PPARGC1A* rs8192678 variant against gestational diabetes is modified by bisphenol A exposure and thyroid-stimulating hormone levels, exhibiting a quasi-U-shaped relationship with BPA and attenuation at higher TSH concentrations. This work exemplifies the paradigm of gene-environment interaction, demonstrating that genetic effects are not fixed but rather context-dependent. At the mechanistic level, Qi and Wang employ integrated gut microbiota and metabolomic profiling to reveal coordinated shifts in specific taxa—particularly Proteobacteria and Enterobacteriaceae—and serum metabolites including carnosine, ornithine, and glycine in obese individuals, establishing a microbiota-metabolite axis that may drive obesity-related inflammation and metabolic dysregulation.

The translational implications of this research are further explored through investigations of novel dietary interventions and their systemic effects. Rodriguez et al., in a retrospective analysis of Sprague Dawley rats, demonstrate that a triple-diet paradigm combining high-fat, high-glucose, and standard chow produces accelerated weight gain through increased caloric consumption driven by dietary variety, offering a practical model for efficient obesity research. Meanwhile, Hu et al. conduct a nationwide population-based study examining eight anthropometric indices and Parkinson's disease, revealing that central obesity measures—particularly a body shape index (ABSI), weight-adjusted waist index (WWI), and conicity index—show stronger associations with PD prevalence than BMI, highlighting the neurodegenerative consequences of visceral adiposity.

Synthesizing the vast and fragmented literature on obesity-diabetes interactions, Liu et al. perform a comprehensive bibliometric analysis mapping research across five major diabetic complications. Their work quantitatively confirms that obesity serves as a central intellectual hub connecting diabetic kidney disease, angiopathy, neuropathy, retinopathy, and foot ulcers, with thematic evolution revealing shifts from foundational associations toward mechanistic pathways (e.g., NF-kappa B), therapeutic interventions (metabolic surgery), and vulnerable populations (children). This macroscopic view complements the molecular and clinical studies in this Research Topic, providing a roadmap for future interdisciplinary collaboration.

Collectively, the articles in this Research Topic advance a vision of obesity research that transcends simplistic energy-balance models. They illuminate the heterogeneity of obesity phenotypes, the plasticity of genetic effects in response to environmental cues, and the systemic nature of metabolic dysfunction that connects adipose tissue to distant organs—from the heart to the brain, from the liver to the placenta. Several cross-cutting themes emerge: first, the imperative to develop and validate ethnicity-specific, multi-dimensional adiposity indices that capture visceral fat distribution and metabolic function; second, the recognition that metabolic health and disease exist on a continuum, with transitions influenced by gene-environment interactions operating across the life course; third, the potential for integrated multi-omics approaches to uncover novel biomarkers and therapeutic targets; and fourth, the urgent need for precision prevention strategies that account for individual variability in genetic susceptibility, environmental exposures, and metabolic phenotypes.

As we look toward future directions, the findings presented here call for prospective studies that track metabolic trajectories from early development through adulthood, randomized controlled trials that test interventions targeting specific metabolic phenotypes, and implementation science that translates these insights into clinical practice. The challenge of obesity and its metabolic consequences will not yield to single solutions, but the collective evidence assembled in this Research Topic illuminates pathways toward more effective, personalized approaches to prevention and treatment. We are grateful to all contributing authors, reviewers, and Frontiers in Nutrition editorial staff who made this Research Topic possible, and we hope these articles will inspire continued investigation into the nutritional, genetic, and environmental determinants of metabolic health.
